# Comparative Transcriptomic Analysis of the Response to Cold Acclimation in *Eucalyptus dunnii*

**DOI:** 10.1371/journal.pone.0113091

**Published:** 2014-11-20

**Authors:** Yiqing Liu, Yusong Jiang, Jianbin Lan, Yong Zou, Junping Gao

**Affiliations:** 1 Department of Ornamental Horticulture, China Agricultural University, Beijing 100193, China; 2 College of Life Science & Forestry, Chongqing University of Art & Science, Yongchuan 402160, China; Cankiri Karatekin University, Turkey

## Abstract

*Eucalyptus dunnii* is an important macrophanerophyte with high economic value. However, low temperature stress limits its productivity and distribution. To study the cold response mechanisms of *E. dunnii*, 5 cDNA libraries were constructed from mRNA extracted from leaves exposed to cold stress for varying lengths of time and were evaluated by RNA-Seq analysis. The assembly of the Illumina datasets was optimized using various assembly programs and parameters. The final optimized assembly generated 205,325 transcripts with an average length of 1,701 bp and N50 of 2,627 bp, representing 349.38 Mb of the *E. dunnii* transcriptome. Among these transcripts, 134,358 transcripts (65.4%) were annotated in the Nr database. According to the differential analysis results, most transcripts were up-regulated as the cold stress prolonging, suggesting that these transcripts may be involved in the response to cold stress. In addition, the cold-relevant GO categories, such as ‘response to stress’ and ‘translational initiation’, were the markedly enriched GO terms. The assembly of the *E. dunnii* gene index and the GO classification performed in this study will serve as useful genomic resources for the genetic improvement of *E. dunnii* and also provide insights into the molecular mechanisms of cold acclimation in *E. dunnii*.

## Introduction

Rapid population increase and the consequent increase in the requirement for different types of paper products, as well as the emphasis on paper as an environmentally friendly packaging material, have led to an increased demand for wood [1]. The imbalance between the supply and demand for forest products is growing. Eucalyptus is an economically important forest tree that grows in tropical and subtropical regions [2], [3]. Eucalyptus trees can be highly productive over a short rotation period, tolerate a wide range of soils and commonly exhibit a straight stem form in those species utilized in production forestry. Furthermore, eucalypts, unlike many trees, do not have a true dormant period and retain their foliage, which enables growth during warm winter periods [4]. Nevertheless, in Eucalyptus plantations, low temperature stress limits their productivity and distribution. When the temperature drops to 8°C or below, Eucalyptus trees would exhibit various symptoms of cold injury due to their inability to adapt to the low temperature [5]. Cold stress also alters the physiological status, such as transient increases in hormone levels (e.g., ABA), changes in the membrane lipid composition, accumulates of compatible osmolytes (such as soluble sugars, betaine, and proline) and increases in antioxidant levels [6], [7]. In contrast, temperate plants can withstand freezing temperatures following a period of low, but non-freezing temperatures, a process called cold acclimation. The mechanisms of cold acclimation have been extensively investigated in *Arabidopsis thaliana* [8] and other important crop species such as maize and barley [9], [10]. Cold stress has been shown to induce changes in physiology and gene expression, and hundreds of cold-responsive genes have been identified so far [11]. However, in tropical and subtropical plants, especially *E. dunnii*, the molecular mechanisms of the cold response are not clear.

The physiological and biochemical changes that occur during plant cold acclimation result primarily from changes in the expression of cold-responsive genes. In general, Cold-responsive genes could be classified into two groups: 1) functional proteins, which directly protect plants against environmental stresses, and 2) regulatory proteins, which regulate the expression of downstream target genes in the stress response [4]. The first group mainly comprises enzymes involved in the biosynthesis of various osmo-protectants, such as late embryo genes is abundant (LEA) proteins, antifreeze proteins, chaperones, and detoxification enzymes[8], [12]. The second group mainly includes transcription factors and protein kinases [12]. The best-characterized transcription factors (TFs) involved in the plant cold response are the class of AP2/ERF (APETALA2/ethylene-responsive element binding proteins), one kind of subfamily was known as CBF/DREB(C-repeat binding factor/dehydration resistance element binding protein), which regulate cold-responsive gene expression by binding to DRE/CRT cis-elements in the promoter region of cold-responsive genes [6], [13]. Changes in the expression of cold-responsive contribute to the differences in plant cold tolerance. For example, *Solanum commersonii* and *S. tuberosum*, which are closely related species that differ in their cold acclimation abilities, exhibit considerable differences in the expression levels of cold-responsive genes [6], [14]. Chen *et al*. found that the activities of some detoxification enzymes, such as catalase (CAT), superoxide dismutase (SOD), peroxidase (POD) and esterase (EST) are increased in response to cold stress, whereas the plant's metabolic activity is decreased [15]–[17]. Some cold-induced genes have been cloned from Eucalyptus plants. For example, four CBF paralogs were previously isolated from *E. gunnii*, and qRT-PCR analysis demonstrated that they exhibited complementary expression profiles in a range of natural standard and cold conditions [18]. Navarro *et al*.found overexpression of *EguCBF1a* or *EguCBF1b* in the cold-sensitive *E. urophylla·E. grandis* hybrid could enhance its freezing tolerance [19].

Given the importance of cold-responsive genes in plant cold tolerance, studying the cold response at the transcription level may be a key step in identifying specific tolerance mechanisms. Next generation sequencing (NGS) provides a high throughput approach for analyzing genes involved a particular process at transcription level. Compared to the traditional sequencing techniques, NGS is more robust and demonstrates greater resolution and inter-lab portability compared to several microarray platforms. NGS could detect millions of transcripts and is beneficial to explore new genes and their expression profiling independent of a reference genome [6], [20], [21]. For example, cDNA libraries for *E. gunnii* have been constructed to identify genes involved in cell protection (such as PCP, Lti6b and metallothionein), LEA/dehydrin accumulation, and cryoprotection [22], [23]. Despite its obvious potential, these next generation sequencing methods have not been applied for *E. dunnii* yet.

The goal of this study was to construct a comprehensive transcriptome to investigate the molecular mechanism of cold tolerance in *E. dunnii*. The plants were exposed to low temperature (4°C) for 0, 3, 6, 12, and 24 h, and the first two expanded leaves below apical bud of *E. dunnii* were collected for high throughput RNA-Seq analysis. Paired-end (PE) reads from the RNA-Seq output were then assembled *de novo* to build an *E. dunnii* transcriptome, which was subjected to a comparative analysis. This analysis provides preliminary global insight into the molecular mechanism of cold tolerance and a good base for future basic research in *E. dunnii*.

## Results

### Physiological changes in *E. dunnii* in response to cold stress

**Figure 1:**
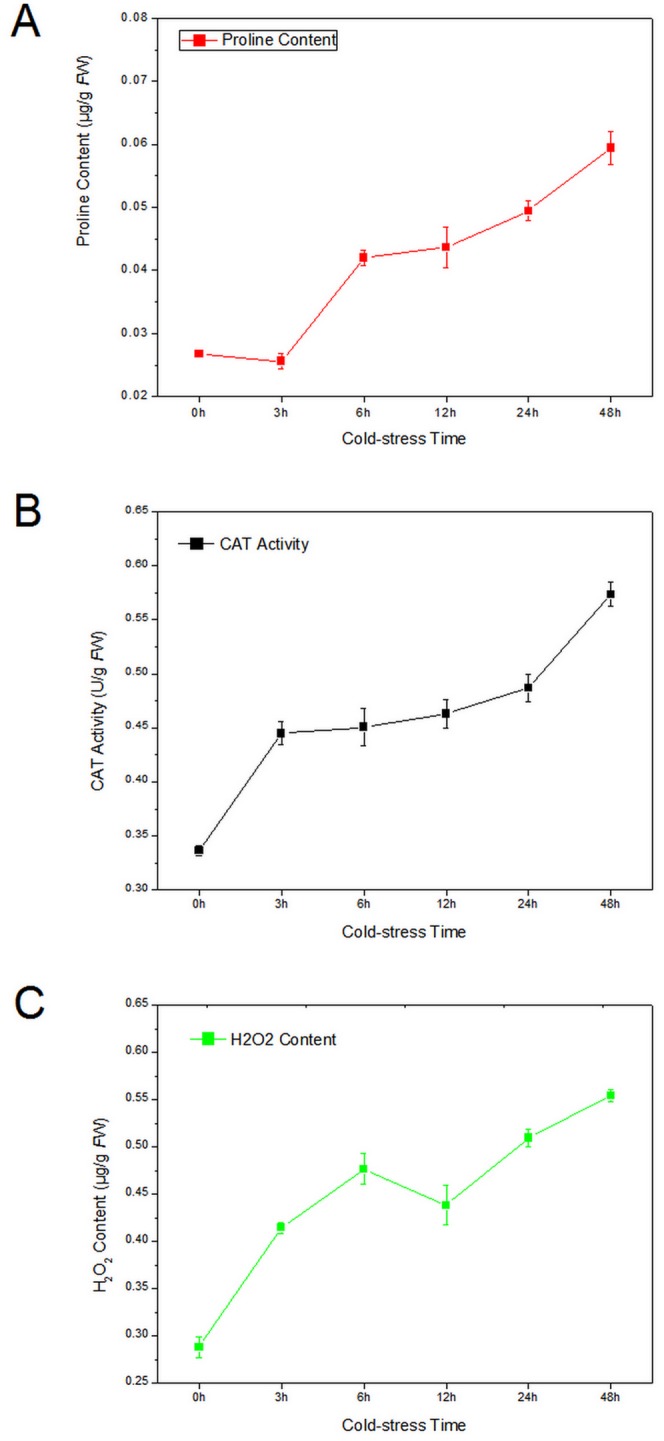
Changes in proline content (A), CAT activity (B) and H_2_O_2_ content (C) under low temperature (4°C) treatment over time.

Firstly, we detected the concentration of proline during the cold treatment (at 4°C) from 0 to 48 h. The concentration of proline decreased slightly from 0 to 3 h, but it increased rapidly as the cold stress prolonging (Fig. 1A). The decrease in proline content at 0 to 3 h might be caused by a transient stress response of *E. dunnii* to the low temperature shock. However, prolonged exposure to low temperature (24 h) resulted in proline accumulation.

Plant cells could accumulate amounts of reactive oxygen species under environmental stress, which result in severe damage of proteins, membrane lipid, DNA and other cellular components [15]. CAT could catalyze the decomposition of hydrogen peroxide to water and oxygen, and it is important in protecting the cell from oxidative damage by reactive oxygen species (ROS) [16]–[17]. The activity level of CAT changed during cold acclimation in *E. dunnii*. We observed an almost 25% increase in CAT activity after 3 h, and a nearly two-fold change after 24 h of cold stress (Fig. 1B). H_2_O_2_ is one kind of ROS molecule. The H_2_O_2_ concentration increased nearly 50% after 3 h, and then continued to increase at a more moderate rate, remaining at high levels until 24 h (Fig. 1C). These results indicated that *E. dunnii* plants are sensitive to the cold stress.

### RNA Sequencing, *de novo* assembly and functional annotation

To study the *E. dunnii* transcriptome in response to cold stress, we transferred plantlets with 10 leaves to a climate-chamber (4°C) and collected the first two expand leaves below apical bud at 0, 3, 6, 12, and 24 h time points, respectively. For the RNA-Seq analysis, we obtained 25,407,247, 24,817,373, 25,703,824, 34,870,702, and 33,846,411 clean paired-end reads, respectively (data not shown).

**Figure 2:**
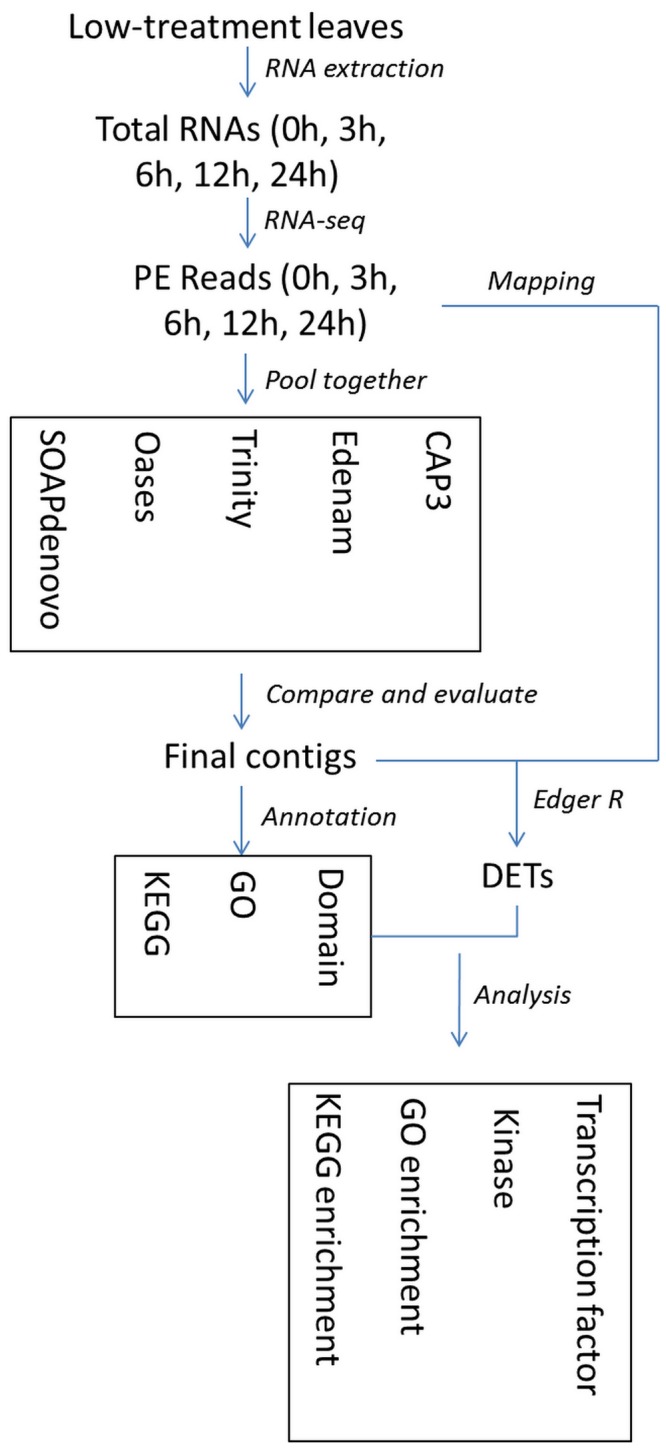
The pipeline for the bioinformatics analysis of the deep sequencing data.

**Figure 3:**
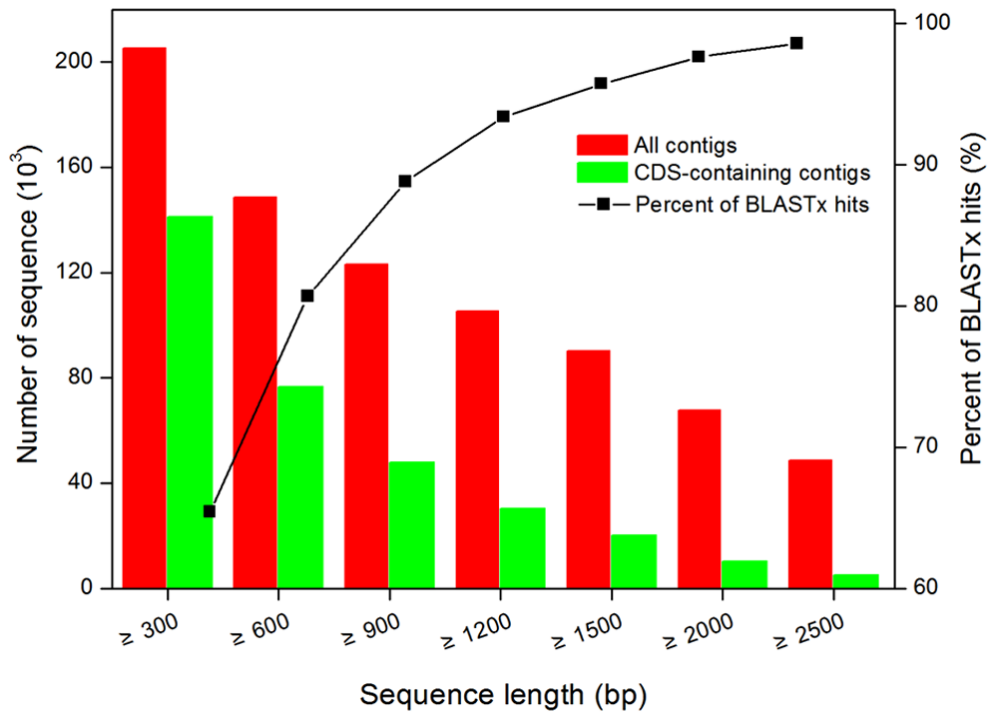
Annotation rate and proportion of long-CDS-containing sequences. A total of 205,325 contigs were used for the BLASTx search. The contig length is indicated on the X-axis. The size distributions of the final assembled contigs (red) and the number of long-CDS-containing contigs (green) are indicated on the left Y-axis. The percentage of BLASTx hits to size-grouped contigs is indicated by the diamond symbol.

To obtain a more reliable and comprehensive transcriptome database, these five libraries were pooled together and then performed the *de novo* assembly. The pipeline for the bioinformatics analysis of the RNA-Seq data is shown in Fig. 2. The parameters of the contig databases assembled by each individual assembler, such as the alignment rate, sensitivity, accuracy and length distribution, were significantly different. Overall, the contig database produced by Trinity was significantly better than those from the other assemblers (Table S1). The optimal contig database contained 205,325 contigs ≥300 bp in length. The average length of these contigs was 1,701.6 bp, the N50 number was 2,827 bp, and the maximum length was 15,965 bp (Table 1). Additionally, there were 148,151 contigs with a length≥600 bp, 105,494 contigs with a length ≥1,200 bp, and 33,700 contigs with a length ≥3,000 bp (Fig. 3). The assembled contigs (≥300 bp) were deposited in the NCBI Transcriptome Shotgun Assembly (TSA) database under the accession number PRJNA208093.

**Table 1:** Total number of reads for each treatment sample, as obtained by Illumina sequencing.

Duration of low temperature (4°C) treatment	Paired-end reads	Total length	Total number of contigs	Average length	N50 of contigs	Alignment rate (%)
0 h	25,407,247	120,616,917	118,761	1,015	1,817	90.3
3 h	24,817,373	179,471,852	149,467	1,200	2,136	91.7
6 h	25,703,824	215,673,491	161,627	1,334	2,343	90.6
12 h	34,870,702	302,614,952	195,733	1,546	2,630	92.4
24 h	33,846,411	217,801,712	160,461	1,357	2,367	91.2
TOTAL	–	349,381,021	205,325	1,701	2,827	94.5

**Figure 4:**
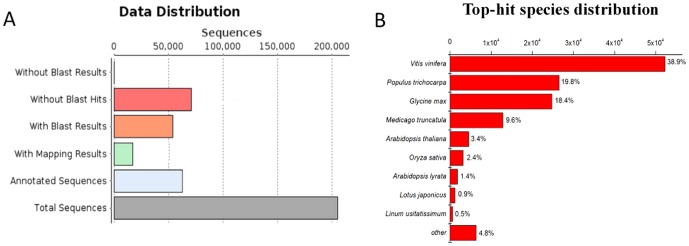
Distribution of the BLASTx results (A) and the top-hit species distribution of the *E. dunnii* transcriptome (B). A total of 205,325 contigs ≥300 bp in length were used for the sequence similarity searches, and 134,358 contigs produced BLASTx results. All of the contigs with BLASTx results were used for the species distribution analysis. Overall, 52,265 (38.9%), 26,602 (19.8%) and 24,721 (18.4%) contigs showed strong similarity to *Vitis vinifera*, *Populus* and *Glycine max*, but only 265 contigs (0.2%) shared the highest homology with Eucalyptus.

Sequence similarity search against the NCBI non-redundant protein database (NR) was conducted using a locally installed BLAST program for functional annotation. Among all the assembled contigs (≥300 bp), 134,358 (65.4%) were annotated with BLASTx hits, matching 80,578 unique protein accessions (Table S2). For contigs longer than 600 bp, 80.9% had BLASTx hits, and for longer than 900 bp, the percentage increased to 88.8% (Fig. 3), indicating that most contigs, particularly the longer contigs, represent protein-encoding transcripts. As the completed genome information of *E. dunnii* was not available at this time, 70,967 contigs (34.6%) had no hits to any known proteins in the Nr database (Fig. 4A), suggesting that these contigs might be non-coding regions or potentially new genes [24].

In addition, among 134,358 contigs with BLASTx results, 52,265 (38.9%), 26,602 (19.8%), and 24,721 (18.4%) showed high sequence similarity to *Vitis vinifera*, *Populus* and *Glycine max*, respectively, but only 265 contigs (0.2%) shared homology with Eucalyptus (Fig. 4B). Alternatively, our results could indicate that *E. dunnii* is more closely to *V. vinifera* than *G. max* or *Arabidopsis* evolutionarily. Interestingly, some other plant transcriptomes, such as *Craterostigma plantagineum* [25] and *Fraxinus* spp. [26], display the same distribution pattern of top-hit species. These results could be simply explained by the number of genes deposited in Nr database. For example, by November 2013, the NCBI database contained 78,045 *V. vinifera* transcripts, 11,4590 *P. trichocarpa* transcripts, and 81,270 *G. max* transcripts, but only 7,146 Eucalyptus transcripts.

### Differential expression between the groups and qRT-PCR validation

**Figure 5:**
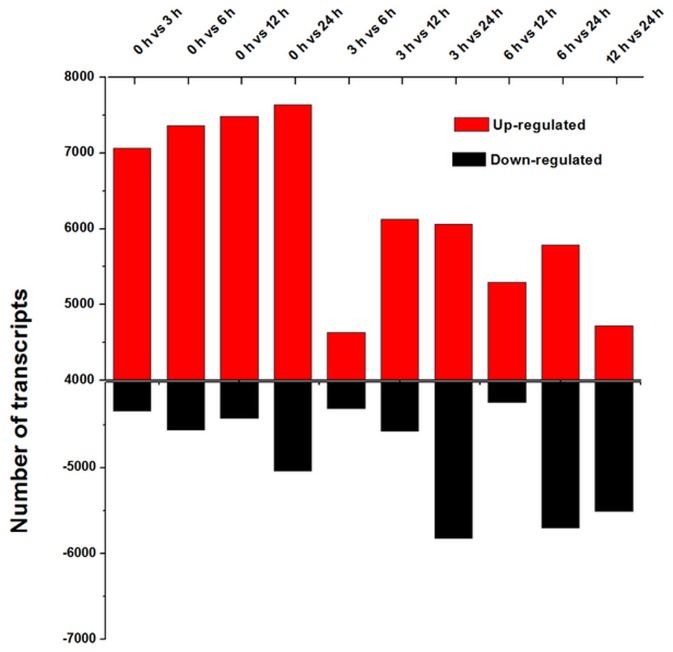
Transcripts that exhibited differential expression pattern. In total, 20,5325 contigs were used for the differential expression analysis, and the differential transcripts were identified by edgeR using the following parameters: FDR ≤0.05 and log_2_fold-change ≥1.

To characterize the digital gene expression profiles of the *E. dunnii* in response to low temperature, we performed a short-read alignment of each library using Perl script provided by the Trinity software package. For samples treated at 4°C for 0, 3, 6, 12, and 24 h, a total of 90.3%, 91.6%, 92.1%, 91.2%, and 91.5% of the reads could be aligned back to the contigs, and 64.2%, 63.8%, 65.1%, 62.7% and 61.5% aligned concordantly exactly once. To eliminate the effect of library size, edgeR (empirical analysis of digital gene expression in R) was used to create an effective library size for each sample. The number of aligned reads per transcript was normalized to FPKM based on an RESM-based algorithm. Differentially expressed transcripts (DETs) with FDR ≤0.05 and log_2_ fold-change (log_2_FC) ≥1 between pairs of samples were identified by edgeR [27]. The edgeR analysis generated 10 DET sets (0 h vs. 3 h, 0 h vs. 6 h, 0 h vs. 12 h, 0 h vs. 24, 3 h vs. 6 h, 3 h vs. 12 h, 3 h vs. 24 h, 6 h vs. 12 h, 6 h vs. 24 h, and 12 h vs. 24 h) with 11,395, 11,908, 11,901, 12,671, 8,935, 10,641, 11,843, 9,531, 11,489, and 10,230 DETs, respectively (Fig. 5). We also found that 7,059, 7,348, 7,479, and 7,636 DETs were up-regulated in the 0 h vs. 3 h, 0 h vs. 6 h, 0 h vs. 12 h, and 0 h vs. 24 h comparison sets, respectively. These results demonstrated that the number of up-regulated DETs increased as the duration of cold stress prolonged.

To validate the expression patterns of each DET obtained from the comparative RNA-Seq studies, we randomly selected 31 transcripts from the annotated DETs for qRT-PCR analysis. Noteworthy, qRT-PCR results are often affected by the choice of reference genes. Previously, a report explored the expression stability of reference genes which are using in gene expression test in Eucalyptus in response to various abiotic stresses by qRT-PCR [28]. The authors found that expression of some genes, such as *PP2A-3/SAND*, *UPL7*, *UBC2* and *GAPDH*, are stable enough in all tested samples, while *ACT2* gene was not stable in response to environmental stimuli as expected. As mentioned in the paper of Cassan-Wang et al., GAPDH is a good choice as reference gene in qRT-PCR assay in Eucalyptus [28]. Therefore,we selected two most commonly used reference genes, beta-actin and GAPDH,because these two genes could be mutual support, mutual correction, and minimize the experimental errors. The results showed that the expression patterns of 25 DETs were compatible with the RNA-Seq analysis (Table S3), suggesting that the differential expression analysis based on high-throughput RNA sequencing produced reliable expression data.

### Gene ontology (GO) and Kyoto Encyclopedia of Genes and Genomes (KEGG) enrichment analysis of DETs

**Figure 6:**
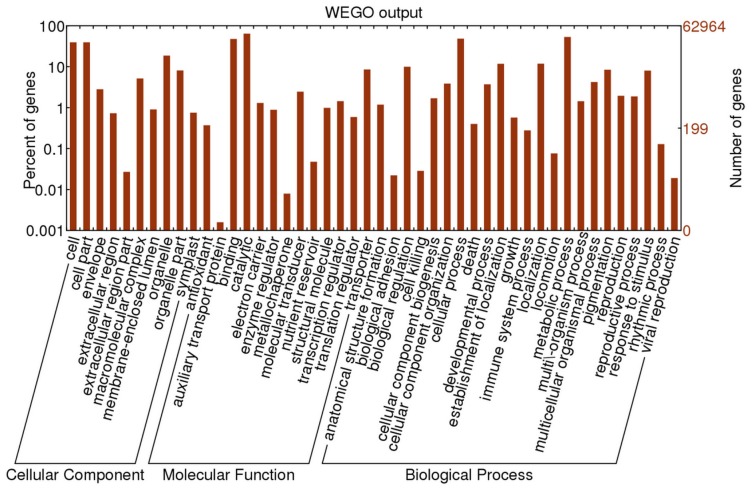
GO assignment of all contigs in the *E. dunnii* transcriptome. The contigs mapped to three main categories: Biological process, Cellular component and Molecular function. The right-hand y-axis indicates the number of annotated contigs.

GO (Gene Ontology) and KEGG (Kyoto Encyclopedia of Genes and Genomes) annotation were applied to the BLASTx results to provide comprehensive functional information for each transcript. In total, we obtained 198,528 GO annotations for 62,965 transcripts and 966 unique Enzyme Codes (ECs) for 28,295 transcripts (Table S2). Among the 62,965 transcripts with GO terms, 34,064 (54.1%) were assigned to the Biological process category, 19,959 (31.7%) to the Molecular function category, and 28,965 (46.0%) to the Cellular component category. In addition, 20,025 (31.8%) unique transcripts were assigned GO terms from all three categories (Fig. 6 and Table S4). To understand the mechanism of the cold stress response in *E. dunnii*, the DETs were subjected to GO and KEGG enrichment analysis. Under the GO category ‘Biological process’, the ‘response to stress’ and ‘translational initiation’ were the most highly enriched terms, with P.ad-values of 0 and 0.02, respectively. Under the category ‘Molecular function’, the ‘quinolinate synthetase A activity’ were the most highly enriched term, with a P.ad-values of 0.04. Under the category ‘Cellular component’, the ‘cell part’ was the most highly enriched term, with a P-value of 8.5E-11 (Table S4). KEGG analysis identified 27,688 contigs with pathway information were involved in 137 KEGG pathways. Among these 137 KEGG pathways, ‘arginine and proline metabolism’ and ‘tropane, piperidine and pyridine alkaloid biosynthesis’ were the two most significantly enriched KEGG pathways (Table S5).

### Cold-responsive transcription factors and protein kinases in *E. dunnii*

Transcription factors and protein kinases are crucial upstream regulators that respond to various biotic and abiotic stresses in plants [29], [30]. In this study, we identified a total of 586 contigs involving in transcription factor activity, which were classified into 65 types of transcription factors, including AP2, bZIP, JmjC, and SRF-TF. In order to verify the expression pattern of these transcription factors, an additional 5 transcripts were selected to carry out qRT-PCR analysis. The results displayed that the expression trend of these transcripts agreed with the results of RNA-seq analysis (Table S3). The START and bzip domains transcription factor families were the largest groups represented in the cold-responsive transcription factors, containing 64 (39 up-regulated and 25 down-regulated) and 62 (43 up-regulated and 19 down-regulated) unique transcripts, respectively. The next largest groups were the UDF (31 up-regulated and 24 down-regulated), AP2 (27 up-regulated and 24 down-regulated) and Sigma70 (24 up-regulated and 9 down-regulated) families (Table S6). In addition, we identified 169 contigs related to protein kinase activity, which were classified into 8 types of protein kinases based on their domains (Table S6).

## Discussion

### Improving *de novo* transcriptome assembly

The most critical step of an RNA-Seq study is the *de novo* assembly, especially for species without genome information [31]–[34]. More and more genomes and/or transcriptomes sequences have been completed due to the development of high-throughput sequencing technologies. However, major published studies on transcriptome *de novo* assembly have typically used a single assembly program [35]–[37]. In this study, we compared the quality of 5 assemblers (Trinity, Osease, SOAP*denovo*, Edena, and Cap3) and then used the optimal combined strategy to construct the *E. dunnii* transcriptome database. When the reads were assembled using Trinity, Cap3, Edena, Oases, and SOAP *de novo*, the N50 (contig ≥300 bp) values were 2,827 bp, 2,551 bp, 1,368 bp, 1,838 bp, and 1,336 bp, respectively (Table S1). Although the accuracy and sensitivity of the contigs assembled by Trinity were the highest compared to the other assemblers, the assembly strategy still needs further optimization to obtain higher accuracy and sensitivity (Table S1). Different assembly software programs used different algorithms, such as the traditional OLC approach of the Edena assembler and the de Bruijn graph approach of the Oases and SOAP *de novo* assemblers [38]. For a particular species, these different algorithms have multiple advantages and disadvantages, which should be taken into account when selecting the most suitable assembler to complete the process of *de novo* assembly in different species. However, neither Trinity nor any other assembler is individually capable of assembling the results satisfactorily. When assembling the sweet potato transcriptome, Tao *et al*. [31] found that only 80% of the reads mapped back to contigs assembled by Trinity, implying that approximately 20% of the reads were not used effectively in the assembly process. In addition, sequencing quality, which is the foundation for obtaining an ideal assembly, should be improved. Xiao *et al*. [38] found that trimming all raw read sequences at the 3′-end and merging the assemblies from different assemblers significantly improved assembly outcome. Some researchers have also suggested that combining data produced by two or more sequencing methods, such as Illumina sequencing and 454 sequencing, could generate a more satisfactory assembly [39]. Combined assemblies use different assembling software and/or different assembling parameters, which means they benefit from the advantages of different software packages.

To date, there have been no standard criteria to evaluate the quality of transcriptome assemblies. Researchers appraise the quality of an assembly mainly by examining the data distribution of the assembly [40], [41]. Besides the data distribution, we assessed the assembly quality using numerous metrics. Due to the lack of genomic resources for *E. dunnii*, we downloaded Eucalyptus genes with full-length from GenBank to use as reference sequences. The overlapping high-scoring segment pairs (HSPs) were only calculated once to determine the sensitivity. For each individual assembly, Trinity achieved higher sensitivity than Cap3, Oases and SOAP *de novo*. However, the final assembly generated by Edenam exhibited the highest sensitivity, which was slightly higher than that of Trinity (Table S1). To calculate the accuracy, we considered all unmatched components to be false positives, and Trinity exhibited a greatest accuracy. Taken together, the results from the above metrics indicate that our final assembly quality is optimal.

### DETs involving in proline metabolism and quinoline alkaloid biosynthesis

Free proline in plant cells can significantly improve cold resistance [42], as it acts as a type of osmotic adjuster that can reduce the cell freezing point and stabilize intracellular water. Furthermore, free proline can also protect the cell from excessive dehydration and lipid peroxidation [42]. The accumulation of proline is frequently associated with whole plant tolerance to chilling and other stresses [6]. In this study, we observed that the free proline content was increased more than two-fold after 48 h cold treatment (Fig. 1), which were consistent with that accumulation pattern in Arabidopsis and cassava [6], [43].

KEGG analysis showed that the ‘arginine and proline metabolism’ pathway was significantly enriched (Table S5) in *E. dunnii* during cold-stress. A total of 576 transcripts were involved in the ‘arginine and proline metabolism’ pathway, with 79 transcripts being up-regulated in response to cold stress at 24 h (Tables S6, S2).

**Figure 7:**
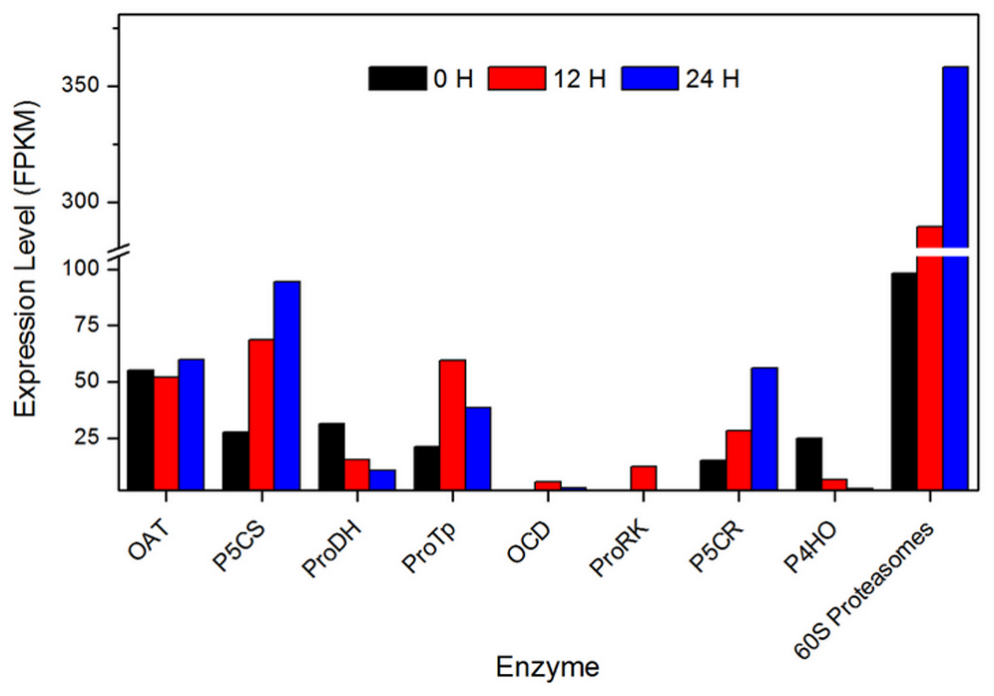
The expression level of some key enzymes involved in the ‘arginine and proline metabolism’ pathway during cold acclimation. Both the up-regulated expression of OAT (ornithine-oxo-acid transaminase), P5CS (pyrroline-5-carboxylatesynthase), ProTp (proline transporter), ProRK (proline-rich receptor protein kinase), P5CR (pyrroline-5-carboxylate reductase), and the down-regulated expression of ProDH (proline dehydrogenase), P4HO (prolyl 4-hydroxylase), OCD (ornithine cyclodeaminase) could result in proline accumulation.

In higher plants, proline can be synthesized via the glutamate (Glu) pathway or the ornithine (Orn) pathway, depending on the initial substrate [44], [45]. P5CS (delta 1-pyrroline-5-carboxylate synthetase), a key enzyme in the Glu pathway, functions as a bifunctional enzyme to transform Glu to GSA [46]. The accumulation of free proline could improve the ability of stress resistance in many plants, which regulated by the expression of *p5cs* [47], [48]. In *E. dunnii*, 3 transcripts were annotated as *p5cs*, and all three transcripts were up-regulated, particularly contig_6788, whose expression increased more than 10-fold when the plants were exposure to low temperature (Fig. 7 and Table S2). This transcriptome result correlated well with the change in free proline content, suggesting that at 4°C, the Glu pathway was activated to increase the free proline content to protect the plant against cold stress. δ-OAT (ornithine-oxo-acid transaminase) is a key enzyme in the Orn pathway that catalyzes the transformation of L-Orn into GSA. Because δ-OAT can catalyze arginine to glutamate, it could be involved in proline synthesis and accumulation [49]. However, we only identified two contigs (contig_6006 and contig_60065) annotated as *δ*-OATin in the *E. dunnii* transcriptome, and neither was up- or down-regulated in response to cold stress (Fig. 7 and Table S2). Based on the expression profiles of these transcripts, we hypothesize that the Orn pathway may play a less important role than the Glu pathway during cold acclimation or that it may represent an alternative pathway for cold acclimation in *E. dunnii*.

Free proline accumulation is affected not only by the proline biosynthesis pathway but also by the proline degradation pathway. Under normal conditions, free proline functions as a feedback regulator to inhibit *p5cs* expression and concurrently induce ProDH (Proline dehydrogenase) gene expression. In contrast to the normal condition, *p5cs* expression is hyperactive during cold acclimation, whereas ProDH expression is inhibited, resulting in the accumulation of more and more free proline in plant cells. In *Arabidopsis* and other plants, proline levels are mainly determined by balance of biosynthetic and catabolic pathways, controlled by P5CS and ProDH genes, respectively [6]. Nanjo *et al*. found that proline degradation was inhibited in *Arabidopsis* transformed with *At*ProDH [43], suggesting that free proline levels increased in leaves.

Secondary metabolism and its products are also involved in the response to various stresses in plants, representing a process that formed over a long evolutionary period [50]–[52]. There is some evidence that secondary metabolic products and environmental factors (biotic and abiotic) are closely linked, as in the case of alkaloids, which play an important role in resisting insects and herbivores via chemical defense mechanism [53]. In addition to ‘arginine and proline metabolism’, the DETs were significantly enriched in ‘quinoline alkaloid biosynthesis’ pathway during cold acclimation, based on KEGG pathway analysis (Table S5). Early in the cold stress period (0–6 h), 40% of transcripts related to quinoline alkaloid metabolism were up-regulated more than 2-fold compared to the 0 h time point (Table S2), including contig_65006 and contig_65485. This suggests that the up-regulation of transcripts in response to low temperatures may play a crucial role in plant stress tolerance. However, when the duration of cold stress exceeded 6 h, the expression levels of these up-regulated transcripts decreased gradually, dropping to their initial levels(i.e., comparable to their expression at 0 h) by 24 h (Table S2). This suggests that there may be a relationship between quinoline alkaloid biosynthesis and abiotic factors, although this relationship may not be as simple and direct as the relationship between the biological environment and chemical defense [54], [55]. Further research is needed to explore this relationship in depth. Many researchers believe that plants produce secondary metabolites such as alkaloids at the cost of slower growth [56], [57]. However, when biotic and abiotic stresses become severe enough to affect their survival, the plants have no choice but to produce some secondary metabolites for protection against such rigorous stress conditions.

### ‘Response to stress’ and ‘translational initiation’ response to low temperature

Under the GO category ‘Biological process’, the terms ‘response to stress’ and ‘translational initiation’ accounted for1.76% of the total 198,528 GOs, but the DETs accounted for 15.4% of the transcripts involved in these GO terms. Additionally, both of two GO terms were significantly enriched in four comparison sets (0 h vs.3 h, 0 h vs. 6 h, 0 h vs. 12 h, and 0 h vs. 24 h) according to the GO enrichment analysis (Table S4). The largest proportion of the ‘Biological process’ terms included the ‘metabolic process’ (30.07%), ‘cellular process’ (27.99%), and ‘biological regulation’ (5.75%) (Table S4), indicating comprehensive changes in *E. dunnii* gene expression before and after the cold stress. However, although only a few transcripts were identified as belonging to ‘response to cold stress’, as up-term of ‘response to stress’, these transcripts represented the most important components that are directly involved in protecting plants from cold stress. A total of 50 transcripts were annotated under this term based on GO categorization, and most were up-regulated in response to low temperature treatment. In particular, 26 transcripts involved in the ‘response to cold stress’ were not expressed under normal conditions but were induced by exposure to low temperature (Table S7 and Fig. S1). ROS scavenging enzymes, including catalase (CAT), superoxide dismutase (SOD), and glutathione transferase (GST), have been demonstrated to play key roles in the removal of ROS [17], [58]–[61]. During exposure to low temperature, the CAT activity was increased (from 0.34 to 0.56 U/g *F*w), which was in accordance with the expression level of the corresponding transcripts in the *E. dunnii* transcriptome (Figs. 1, and 7). Although the expression of peroxidases such as CAT and SOD increased significantly as the duration of cold exposure increased, these enzymes were still unable to completely clear the increased levels of H_2_O_2_, resulting in a significant increase in the amount of H_2_O_2_ during cold acclimation (Fig. 1). In this study, we found that some genes (e.g., MAP kinase and TCH2; Table S2) that are known to be involved in the response to other stresses (including salinity, heat and drought) in other plants are also involved in cold acclimation, which could support the hypothesis that the same gene have different functions in different plants.

The GO term ‘translational initiation’ was enriched in response to cold acclimation. A total of 254 transcripts were annotated under in this term, and 89 exhibited a greater than two-fold change in expression during the low temperature treatment (Table S8). Translation initiation in eukaryotes depends on many eukaryotic initiation factors (eIFs) that stimulate both the recruitment of the initiator tRNA, Met-tRNAiMet, and mRNA to the 40S ribosomal subunit and the subsequent scanning of the mRNA for the AUG start codon [62]–[64]. The largest of these initiation factors, the eIF-3 complex, organizes a web of interactions among several eIFs that assemble on the 40S subunit and participate in the different reactions involved in translation [62], [65]. In plants, eIF-3plays the role of the central protein and interacts with many other translation initiation factors, such as eIF-4F, eIF-4G, eIF-4B, and eIF-1A [66]. Among the 89 contigs we identified that were annotated as ‘translational initiation’, 18 containedeIF-3 (eukaryotic translation initiation factor 3), and almost all were up-regulated during cold acclimation (Table S8). Daniel *et al*. [67] found that the expression level and phosphorylation state of these factors described above is subject to alteration during development, environmental stress (e.g., heat shock, and starvation), or viral infection. Tuteja [68] evaluated the roles of translation initiation, transcription factors, protein kinases, free proline, and reactive oxygen species in plant stress tolerance and found that these factors typically have synergistic effects in response to stress in plants. We also found that some transcripts encoding transcription factors, protein kinases (Table S6), translation initiation factors and antioxidant enzymes were up- or down-regulated in *E. dunnii* during cold acclimation, suggesting that the plant response to cold acclimation is a complex and global process.

### Cold-responsive transcription factor genes in *E. dunnii*

In *Arabidopsis*, at least 5 transcription factor families have been reported to be involved in the cold stress response process, including AP2-EREBP, MYB, NAC, bHLH and WRKY family [29]. Wang *et al*. found there were many families of transcription factor, such as bHLH family, MYB family, WRKY family, NAC family and so on, responding to cold acclimation in *C. sinensis* [16]. Meanwhile, An *et al*. identified 6 AP2-EREBP and 5 Myb transcription factors participated in the process of cold stress in treated cassava [6]. In our study, many transcripts were annotated as AP2 transcription factor based on the domain analysis. Among theses, 27 transcripts were up-regulated and 24 down-regulated under cold stress (Table S6). In present work, we tested 5 AP2 TF genes by qRT-PCR and found four were up-regulated, one was down-regulated during cold-stress, and the changing trend of the two methods was accordant (Table S3). The AP2-EREBP family plays a major role in the early stages of the cold response and is the major regulator that functions in activating cold-regulated effectors in Arabidopsis and other plants [69], [6]. In Eucalyptus plant, the CBF proteins, belonging to A-1 subfamily of ERF/AP2 TF family has been reported involved in response to cold stress in *E. gunnii* and *E. globules* [19], [70]–[72].

Besides the AP2 family, the bZIP family has also been demonstrated to be involved in the cold response in *Arabidopsis* and *C. sinensis* [69], [16]. In this study, we found that bZIP family was the most enriched TF family, containing 62 genes (43 up-regulated and 9 down-regulated). Differential expression of bZIP TFs implies that other environmental or hormonal pathways may be involved in cold response in *E. dunnii*.

In addition, four novel transcription factor families (JmjC, SRF-TF, and Sigma70-like) were also identified. Although their homologous genes in other plant species have not yet been reported in response to cold stress, the expression level of these genes were markedly changed before and after cold stress, suggesting they might be specific to *E. dunnii* or attractive targets for further functional characterization in plant.

## Materials and Methods

### Plant materials

*Eucalyptus dunnii* was used in this study. The plantlets of *E. dunnii* with 10 leaves were grew in a climate-chamber, with a temperature of 25°C, 200 µEm^−2^s^−1^ illumination and a 14/10 h light/dark photoperiod. After eight weeks, the plants were moved into another climate-chamber with a temperature of 4°C and 200 µEm^−2^s^−1^ continuous illumination for cold-stress. For physiological measurement, we harvested the first two expanded leaves of these plantlets at 0, 3, 6, 12, 24 and 48 h after cold treatment, respectively. For RNA-seq, leaves from 6 plants treated by 0, 3, 6, 12, and 24 h were mixed for RNA isolation and sequencing. For test of physiological changes, leaves of plants treated by all time points were used. The harvested leaves were immediately frozen in liquid nitrogen for use.

### Analysis of physiological parameters

The proline content of the leaves was analyzed using a free proline ELISA kit (Omega, Georgia, USA) according to the manufacturer's instructions. The CAT activity and H_2_O_2_ content were measured using a CAT ELISA kit (Omega, Georgia, USA) and a H_2_O_2_ ELISA kit (Omega, Georgia, USA), respectively. All measurements were performed on the platform of Epoch-ELIASA (Shmadzu, Tokyo, Japan), and all analysis were repeated three times in this study.

### RNA extraction, library construction and RNA sequencing

Total RNA was isolated from the leaves by using Trizol reagent (Invitrogen, CA, USA) according to the manufacturer's instructions, and the RNAwas treated with RNase-free DNase I (TaKaRa, Dalian, China). The purity, concentration and RNA integrity number (RIN) were determined using a SMA3000 and/or Agilent 2100 Bioanalyzer. The total RNA was then sent to Beijing Genomics Institute (BGI) -Shenzhen (Shenzhen, China) for RNA sequencing.

More than 20 µg of total RNA extracted from each group of plants exposed to low temperatures (n>3) was used to construct the cDNA libraries. First, the polyadenylated RNAs (mRNAs) were purified and retrieved using magnetic beads coated with a poly-T oligo. These mRNAs were then mixed with fragmentation media and fragmented. The fragmented mRNAs were subjected to reverse transcription using reverse transcriptase and random primers. The second-strand cDNA synthesis was performed using DNA polymerase I and RNase H. Finally, the resulting dscDNAs were repaired by adding a single ‘A’ base, and specific Illumina adapters were ligated to the repaired ends. Fragments of approximately 200 bp in size were purified and retrieved from the gels. To construct the fragmented cDNA library, these fragments, which served as the template, were enriched by PCR using two primers that annealed to the ends of the adapters. The cDNA libraries constructed above were sequenced using an Illumina Hiseq2000. The PE read information and quality values were generated using the Illumina sequencing-by-synthesis, image analysis and base-calling procedures.

### *Denovo* assembly and functional annotation

Sequencing quality was assessed using fastQC software [http://www.bioinformatics.bbsrc.ac.uk/projects/fastqc/], and the PE reads were *de nov*o assembled by five different assemblers: the Trinity software package (v2013-02-25) [73] with default parameters, the Oases software package (v0.1.21) [74] with a different K-value, the Edenam software package (v2013-07-15) [75] with a different M-value, the SOAP *de novo* software package (v2013-07-15) [76] with different K- and P-values, and the Cap3 software package (v12.07.21) [77] with default parameters. To evaluate the quality of the assemblies produced by the different assemblers, the PE reads were aligned back to the contigs assembled by a different assembler using Bowtie2 software (v2.0.0) [78], and the alignment rate was calculated. Subsequently, we analyzed the length distribution information of these contigs, such as the N50 number, average length, max length and total contig number, using common Perl scripts. Due to the lack of genomic information for Eucalyptus, 535 Eucalyptus sequences containing complete CDSs were downloaded from GenBank [http://www.ncbi.nlm.nih.gov/] and used as reference sequences to calculate the sensitivity and accuracy. Furthermore, we analyzed the best candidate coding sequence (CDS) for each contig from different assemblers and obtained the ratios of long CDS-containing transcripts to contigs with corresponding lengths.

All of the contigs (≥300 bp) produced by the Trinity software package were subjected to a similarity search against the NR database downloaded from GenBank utilizing local NCBI-BLAST software (v2.2.28+). The BLASTx searches were performed using a threshold E-value of <10^−3^, max_target_seqs of 5, and an xml output file format. The BLASTx results were imported into Blast2GO software (v2.6.7) [79], and local functional annotation was performed. Enzyme codes, gene ontology (GO), and Kyoto Encyclopedia of Genes and Genomes (KEGG) pathways were retrieved from the KEGG web server (http://www.genome.jp/kegg/) [80]. GO classification [81] was performed using the WEGO program (http://wego.genomics.org.cn/cgibin/wego/index.pl) [82].

### Differential expression profiling and enrichment

To investigate the expression level of each transcript at the five treatment time points, the PE reads for each sample were aligned back to the optimal assembly result (assembled by the Trinity assembler) using Perl scripts provided by the Trinity software package. Using these scripts, we obtained the digital expression levels of each transcript and normalized these data with a RESM-based algorithm to obtain the FPKM (Fragments per Kilobase per Million Mapped Fragments) values of each transcript. Based on the normalized expression profiles, the effect and bias introduced by library size and/or RNA composition were eliminated using edgeR [83], and significant differentially expressed transcripts (DETs) were identified with a P.ad-value ≤0.05 and log_2_ fold-change (log_2_ FC) ≥1.

The DET enrichment analysis was performed using the common Perl and R scripts. We first counted the number of transcripts involved in each KEGG pathway from the Trinity assembled contigs and/or DETs. Based on the transcript numbers in the contig database and DETs, we determined the enriched KEGG pathway. Then, the P-value was adjusted by the Bernoulli equation, and a P.ad-value<0.05 was the threshold value for significant enrichment results. We applied a similar approach for the GO enrichment analysis.

### Expression level verification

To verify the reliability and accuracy of the NGS-based expression level analysis, we randomly selected 31 transcripts from the contig database and evaluated the expression profiles among the five samples using quantitative real-time PCR. The primers for these transcripts are listed in Table S5. The first-strand cDNA was synthesized from 500 ng of total RNA using oligo (dT), random hexamers, and Moloney murine leukemia virus (M-MLV) reverse transcriptase (Invitrogen, CA, USA) according to the manufacturer's instructions. The real-time PCR was performed using the IQ5 Real-Time PCR System (Bio-Rad, CA, USA) in a total volume of 20 µL containing 100 ng of cDNA template, 1× SYBR *Premix Ex Taq* ^TM^II (Perfect Real Time, TaKaRa), and 400 nM of each primer. Serial dilutions of each cDNA were used to generate a quantitative PCR standard curve to calculate the corresponding PCR efficiencies. The PCR conditions were as follows: initial denaturation at 95°C for 30 s, followed by 40 cycles of denaturation at 95°C for 5 s, primer annealing at 60°C for 30 s, and DNA extension at 72°C for 30 s. Two most commonly used reference genes, beta-actin and GAPDH, were selected for internal controls. Three biological replicates were used, and melting curve analysis was performed to check the amplification specificity. The relative expression levels were calculated using the BIO-RAD IQ5 standard edition Optical System software (version 2.1) and a normalized expression (ddCt) model.

## Supporting Information

Figure S1 **Differences of ‘response to stimulus’ (A), ‘response to cold’ (B), ‘transcription factor activity’ (C) and ‘kinase regulator activity’ (D) between each pair of samples.** Overlap examinations were performed based on the resulting gene lists of four comparisons by VENNY. Overlap among four groups, D0 vs D3 (blue), D0 vs D6 (yellow), D0 vs D12 (yellow) and D0 vs D24 (red), were shown here.
(DOCX)

Table S1 **The characteristics of contig databases assembled by different assembler.**
(DOCX)

Table S2 **Sequence annotations of** ***E. dunnii*** **transcripts and gene expression profiling of five samples.**
(XLSX)

Table S3 **Comparison of expression patterns between RNA-Seq expression and qRT-PCR.**
(XLSX)

Table S4 **GO classification of** ***E. dunnii*** **teanscriptome and differentially.**
(XLSX)

Table S5 **KEGG classification of** ***E. dunnii*** **teanscriptome and differentially expressed transcripts indentified between each pairs comparisons.**
(XLS)

Table S6 **Transcription factor and kinase of** ***E. dunnii*** **response to low-temperature stress.**
(XLSX)

Table S7 **Expression patterns of some transcripts involved in 'Response to cold'.**
(XLSX)

Table S8 **Expression patterns of some transcripts involved in 'translational initiation'.**
(XLSX)
