# Supplementary material for: Comparative Transcriptomic Analysis of the Response to Cold Acclimation in Eucalyptus dunnii
Source: PLoS One. 2014 Nov 20;9(11):e113091. doi: 10.1371/journal.pone.0113091 (PMC4239045; doi:10.1371/journal.pone.0113091)

**Fig. S1** Differences of ‘response to stimulus’ (A) , ‘response to cold’ (B) , ‘transcription factor activity’ (C) and ‘kinase regulator activity’ (D) between each pair of samples. Overlap examinations were performed based on the resulting gene lists of four comparisons by VENNY. Overlap among four groups, D0 vs D3 (blue) , D0 vs D6 (yellow) , D0 vs D12 (yellow) and D0 vs D24 (red) , were shown here.


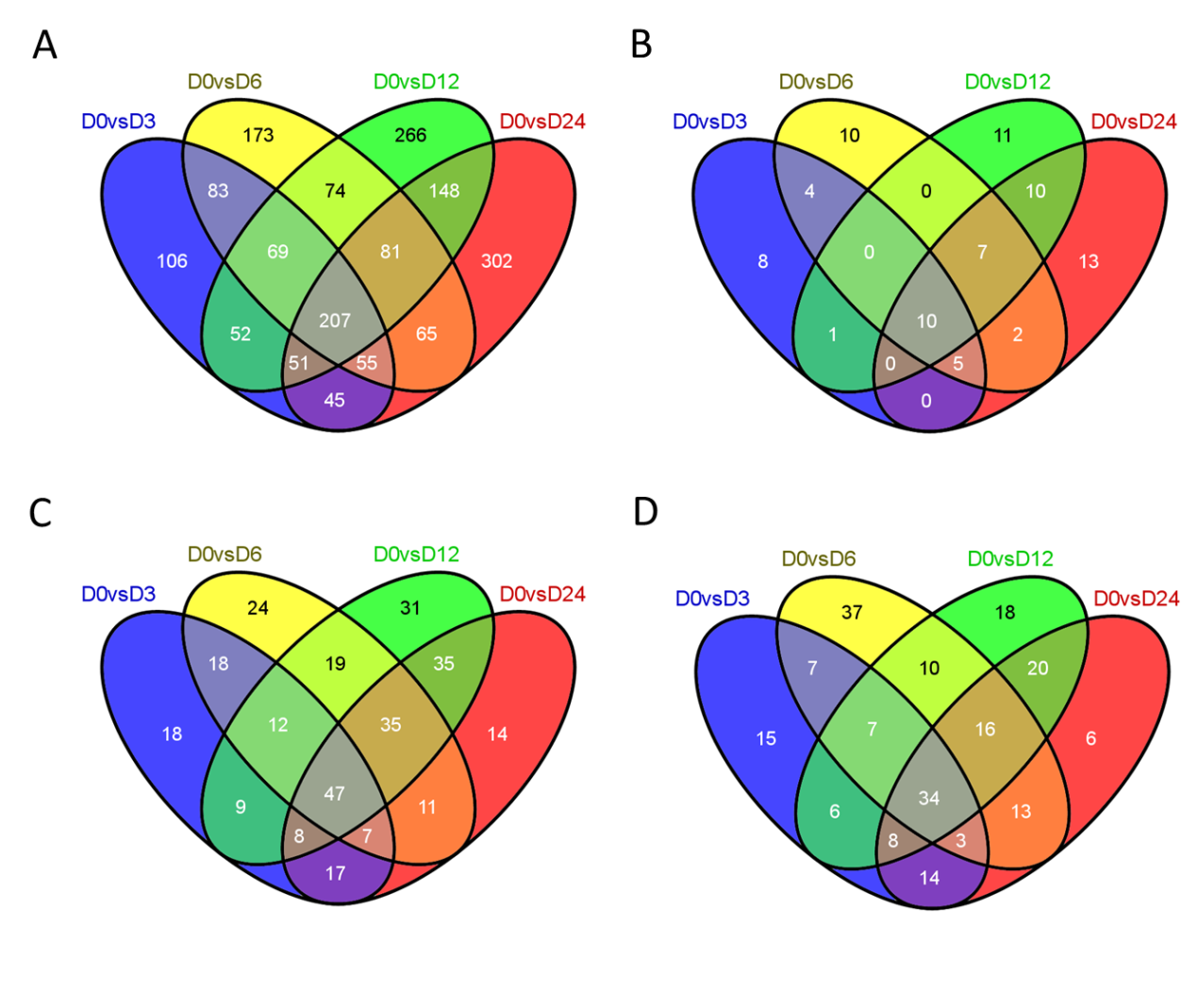

Supplement: Figure S1 — Differences of ‘response to stimulus’ (A), ‘response to cold’ (B), ‘transcription factor activity’ (C) and ‘kinase regulator activity’ (D) between each pair of samples. Overlap examinations were performed based on the resulting gene lists of four comparisons by VENNY. Overlap among four groups, D0 vs D3 (blue), D0 vs D6 (yellow), D0 vs D12 (yellow) and D0 vs D24 (red), were shown here. (DOCX) [file pone.0113091.s001.docx]
