# Supplementary material for: Comparative Transcriptomic Analysis of the Response to Cold Acclimation in Eucalyptus dunnii
Source: PLoS One. 2014 Nov 20;9(11):e113091. doi: 10.1371/journal.pone.0113091 (PMC4239045; doi:10.1371/journal.pone.0113091)
Supplement: Table S1 — The characteristics of contig databases assembled by different assembler. (DOCX) [file pone.0113091.s002.docx]

Tab. S1 The characteristics of contig databases assembled by different assembler.

| Assembler | No. of transcripts | | | N50 (bp) | Average length (bp) | Total length (M bp) | Maximal length (bp) | Aligned rate  (%) | Accuracy | Sensitivity |
| --- | --- | --- | --- | --- | --- | --- | --- | --- | --- | --- |
|  | ≥300 bp | ≥600 bp | ≥1200 bp |  |  |  |  |  |  |  |
| CAP3 | 30104 | 25008 | 58729 | 2551 | 1608.4 | 196.55 | 14985 | 63.4 | 0.82 | 0.62 |
| OasesK27 | 71786 | 45149 | 68463 | 1699 | 1183.8 | 265.19 | 13601 | 83.5 | 0.70 | 0.48 |
| OasesK29 | 64236 | 49636 | 77927 | 1838 | 1292.1 | 274.13 | 13601 | 79.6 | 0.72 | 0.56 |
| Edenam55 | 70283 | 32734 | 40486 | 1368 | 953.7 | 231.81 | 82704 | 76.2 | 0.53 | 0.68 |
| Edenam59 | 79137 | 36161 | 32400 | 1254 | 933.2 | 220.98 | 83196 | 74.8 | 0.57 | 0.71 |
| SOAPdenovoK45p8 | 89382 | 49071 | 50341 | 1336 | 886.5 | 318.23 | 11241 | 82.2 | 0.55 | 0.54 |
| SOAPdenovoK47p4 | 107150 | 43992 | 44371 | 1150 | 809.7 | 285.51 | 10253 | 75.6 | 0.56 | 0.51 |
| Trinity | 57174 | 42657 | 105494 | 2827 | 1701.6 | 349.4 | 15965 | 94.5 | 0.78 | 0.67 |
